# Predictors of Depression among Individuals Receiving the Basic Livelihood Security Program Benefits in Korea: A Study Based on the Sixth and Seventh Korea National Health and Nutrition Examination Survey (2013–2018)

**DOI:** 10.3390/ijerph20010194

**Published:** 2022-12-23

**Authors:** Heejung Choi, Jaelan Shim

**Affiliations:** 1 Department of Nursing, Uiduk University, Gyeongju 38004, Republic of Korea; 2 College of Nursing, Dongguk University, Gyeongju 38066, Republic of Korea

**Keywords:** basic income security, depression, socioeconomic status

## Abstract

The purpose of this study is to verify the relationship between oral health behaviors and depression and influencing factors on depression to establish strategies that can contribute to improvement of mental health problems such as depression in vulnerable social class recipients. This study is a descriptive correlation study conducted on basic livelihood recipients over the age of 19 who responded to the 6th~7th (2013~2018) National Health and Nutrition Examination Survey. In this study, a total of 2749 people who met the criteria for subject selection were included in final analysis after requesting and downloading their raw data from the National Health and Nutrition Examination Survey through the consent process mandated by the Korea Centers for Disease Control and Prevention. Of the 2749 subjects, 279 were diagnosed with depression, accounting for 10.1% of the total. The collected data were analyzed using *t*-tests and chi-squared tests, and factors affecting depression were analyzed by logistic regression analysis. Our results showed that the factors affecting depression were 1.74 times for men (95% CI = 1.29–2.24), 1.37 times for older people (95% CI = 1.01–1.87), and 1.66 times for low education (95% CI = 1.21–2.27). Subjects with impairment in daily activities had 1.89 times (95% CI = 1.43–2.52) higher risk. Subjects with moderate physical activity and subjects with economic activity showed a lower risk (95% CI = 0.30–0.73) and 0.52 times (95% CI = 0.30–0.72), respectively, than subjects who did not. We confirmed that the probability of being diagnosed with depression decreased, and the perceived health status was 0.36 times lower (95% CI = 0.22–0.61) than those with good status. Therefore, it is necessary to prepare countermeasures that reflect various aspects in consideration of not only age and gender, but also daily life and emotional state when establishing policies for vulnerable classes such as recipients of basic livelihood.

## 1. Introduction

Health disparity across various socioeconomic statuses (SES) has emerged as a serious public health issue not only in developed countries [1], but also in developing countries, such as South Korea [2]. From 1 October 2000, the National Basic Livelihood Security Program in Korea replaced the Livelihood Protection System that had been implemented in 1961 and guaranteed a minimum income of 930,000 KRW for a four-member household [3]. The Livelihood Protection Act enacted in 1962 delineated national social security policies, and a basic social security system was established and maintained without strict criteria for eligible beneficiaries. In 1999, the National Basic Livelihood Security Act was enacted, and was enforced from October 2000 [3]. The Basic Livelihood Security Program (BLSP) is a program that provides living expenses, health care, educational support, living arrangements, and other similar types of support for people with low income. Approximately 1.63 million people, equivalent to about 3.2% of the total population, received these benefits in 2016 [4].

The Speenhamland system of the United Kingdom, the Family Assistance Plan of the United States, and the BLSP of South Korea are systems that were implemented in different eras and different countries, but they share the common feature of providing basic income security to the vulnerable socioeconomic class [5].

Economic poverty among BLSP-benefit recipients was found to increase mental health problems and the severity of depression. While the mental health of public welfare recipients has been extensively researched previously, and since the Welfare Reformation in 1996 in the United States, not many studies have examined depression in welfare beneficiaries in particular. Thus, there is a need for further research on the mental health issues affecting welfare recipients [5].

Depression is a mental disorder characterized by a sad mood, low self-esteem, and loss of interest in life. It is a serious disorder that has an impact on overall physical and oral health by disrupting one’s mood and hindering activities of daily living (ADLs), such as working, playing, eating, and sleeping [6,7]. The number of patients seeking medical attention for depression rose markedly, by 30%, in 2020 as compared to that in 2016, with individuals aged 20–29 years (16.9%) most affected, followed by individuals aged 60–69 years (15.8%) [8]. These statistics highlight the importance of continued research on the changes in the prevalence of depression by age. In particular, BLSP-benefit recipients are at an increased risk for mental health problems, such as depression, as compared to the general population, due to economic stress and chronic financial problems [8]. In fact, a study that compared mental health between a poverty group and the general population reported that approximately 26% of the poverty group suffered from mental health problems, such as depression [9]. Additionally, anxiety disorders, the most common comorbidity associated with depression, these anxiety symptoms often precipitate the onset of depressive episodes [10]. According to the 2022 report of Ministry of Health and Welfare of South Korea [11], compared to the situation before the corona pandemic, the prevalence of depression of BLSP-benefit recipients increased about 5 times due to the prolonged COVID-19, there is an urgent need for intervention for depression. Furthermore, depression may lead to suicide in severe cases, early detection is crucial, as are systematic measures to promote the treatment of patients with depression after diagnosis.

Oral health disparities are also evident across socioeconomic classes. A previous study reported that dental caries, periodontal diseases, and tooth loss were more prevalent in adults with low SES and that socioeconomic factors are strongly associated with oral health disparity [12]. This suggests that objective oral health status and oral health behaviors are worse in those with lower SES, and that people of low SES are predicted to have worse oral health management and access to dental care services. In addition, the low-SES population have lower dental health service utilization and greater inequity in dental health service utilization [13]. Therefore, socioeconomic inequality leads to gaps in oral health behaviors, further exacerbating oral health inequity.

Oral health is the foundation of building a good quality of life and maintaining health from young adulthood to middle adulthood and older adulthood [14]. Of various oral functions, chewing function not only affects nutrient intake, but also one’s emotions as well as economic activity, social activity, and learning activity. Masticatory problems can undermine individuals’ motivation to participate in various activities [15]. Oral health status in particular has been strongly linked to depression, and perceived oral health has been reported as a predictor of depression [16]. Park, Wee, and Kim [17] reported a higher prevalence of depression in women brushing their teeth less than 3 times a day, showing a negative correlation between lifestyle practices and depression. In addition to biological and socioeconomic factors, individuals’ lifestyle practices, educational environment, and psychosocial factors may influence oral health [13]. Moreover, oral health behaviors affect oral health but have been strongly associated with SES [18].

Although many prior studies have attempted to identify the predictors of depression among older adults, middle-aged adults, young adults, and the general population [19,20,21,22], few study to date has investigated the association between oral health behaviors and depression. Similarly, few study has investigated the predictors of a depression diagnosis among BLSP-benefit recipients using large-scale data.

Thus, this study investigated the association between oral health behaviors and depression and the predictors of depression in BLSP-benefit recipients, to present foundational data for improving mental health problems, such as depression, in the socially vulnerable BLSP-benefit recipients.

## 2. Methods

### 2.1. Study Design

This study was a descriptive correlational study aiming to examine the association of oral health behavior with depression, and to identify the predictors of depression in BLSP-benefit recipients.

### 2.2. Study Population

The target population of Korea National Health and Nutrition Examination Survey (KNHANES) comprises non-institutionalized Korean citizens residing in Korea. KNHANES is composed of three component surveys: a health interview, health examination and nutrition survey. The health interview and health examination are performed by trained medical staff and interviewers at the mobile examination center. The health interview questionnaire consists of household and individual components [23].

We requested the raw data of the sixth and seventh (2013–2018) KNHANES, as per the Korea Disease Control and Prevention Agency (KDCA) protocol and downloaded the data for analysis.

**Figure 1:**
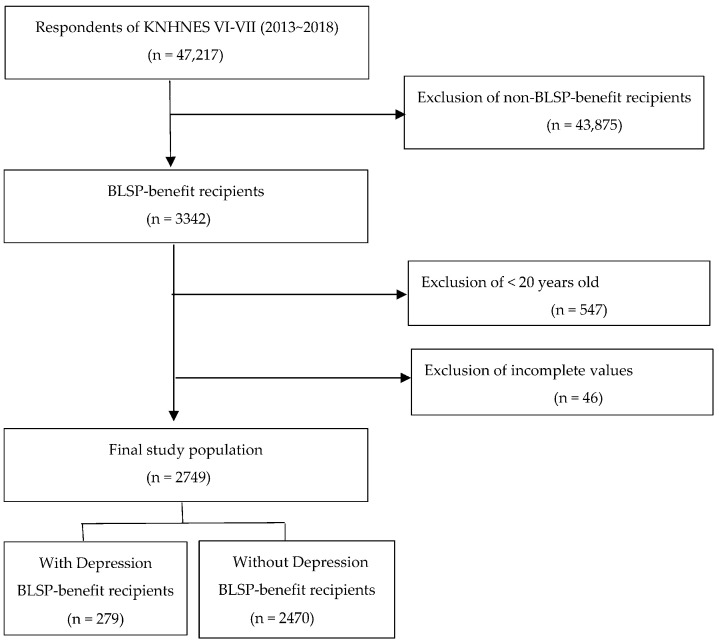
Flow chart of subjects.

The study population comprised 3342 adults aged 19 years and over who received BLSP benefits and responded to the sixth and seventh (2013–2018) KNHANES. Of these, 2749 individuals who responded to all survey items and had no missing data were selected for the final analysis (Figure 1).

### 2.3. Instruments

#### 2.3.1. General Characteristics

With reference to the literature [24,25], general characteristics that were identified as risk factors for oral health and mental health, namely sex, age, marital status, education level, ADLs, moderate physical activity, occupation, smoking, and drinking, were examined.

#### 2.3.2. Oral Health Practices

Oral health practices included oral health checkups in the past year, use of oral hygiene products (i.e., floss, interdental brush, oral rinse solutions, and others, such as a waterpik or tongue cleaner), dental preventive treatment, and daily tooth brushing frequency.

#### 2.3.3. Depression Score: Patient Health Questionnaire-9

The Patient Health Questionnaire (PHQ) is a self-reporting questionnaire developed to help detect and diagnose a few mental disorders commonly encountered in primary care settings. It is the most widely used instrument in clinical and research settings [26]. The PHQ-9 is the depression module of the PHQ [27]. It was translated and adapted into Korean by Han et al. [28]. The scale assesses nine factors (i.e., anhedonia, depressed mood, sleep changes, fatigue, changes in appetite, feelings of guilt or worthlessness, diminished concentration, restlessness or sluggishness, and suicidal ideation). The questionnaire asks the following: “Over the past 2 weeks, how much have you been bothered by the following problems?” Each item is rated on a scale consisting of 0 (not at all), 1 (several days), 2 (more than half the days), and 3 (nearly every day). The highest possible score for each item is 3, with a total score of 27 for the entire scale. The scores were interpreted as “minimal” (0–4), “mild to moderate” (5–14), and “severe” (≥15). A depression diagnosis was classified as yes or no. Cronbach’s α of the Korean version of the PHQ-9 was 0.86 at the time of adaptation [26].

### 2.4. Ethical Considerations

The KNHANES raw data only contain de-identified information, in accordance with the Personal Information Protection Act and Statistics Act. The KNHANES is conducted after review and approval by the KDCA institutional review board (2013-12EXP-03-5C). The official KNHANES data are available for public use. We downloaded the raw data from the KNHANES website (https://knhanes.cdc.go.kr/, accessed on 2 May 2021) after reading the “Rules for KNHANES raw data disclosure and use”.

### 2.5. Data Analysis

The collected data were analyzed using the SPSS for Windows v 25.0 (IBM Inc., Armonk, NY, USA) software as follows: Differences in participants’ general characteristics according to depression were analyzed, using number and percentage, or mean and standard deviation, and the chi-square test. Differences in oral health practices according to depression were analyzed with chi-square tests. Predictors of depression were analyzed using multiple logistic regression. Nominal variables were dummy-coded.

## 3. Results

### 3.1. General Characteristics of BLPS-Benefit Recipients according to Depression

Table 1 shows the general characteristics of BLSP-benefit recipients according to the diagnosis of depression. Of 2749 participants, 279 (10.1%) were diagnosed with depression. The mean age of the participants was 56.26 (SD, 18.02) years.

Age differed significantly according to depression. The majority of individuals diagnosed with depression were female (*n* = 204, 73.1%), married (*n* = 238, 85.3%), and had an elementary school education or less (*n* = 128, 45.9%). From the entire study population, 1475 (53.7%) had an elementary school education or less, and 1781 (64.8%) were not economically active. A total of 1972 participants (71.7%) had no problems with ADLs. Among individuals diagnosed with depression, the majority (75.6%) were not economically active, had no problem with ADLs (57.7%), and did not engage in moderate-intensity physical activity (90.0%). Smoking and drinking status and perceived oral health status did not significantly differ according to depression. Most of the participants diagnosed with depression (63.4%) had poor self-rated health.

The percentage of participants with PHQ-9 scores indicating mild to severe depression (5–15) was 36.2% (*n* = 101) in the depression group and 11.8% (*n* = 292) in the no-depression group (Table 1).

### 3.2. Differences in Oral Health Practices according to Depression

Table 2 shows the differences in oral health practices according to the diagnosis of depression. The percentage of participants who received an oral health checkup in the past year significantly differed between the depression (24.7%) and the no-depression group (18.7%) (χ^2^ = 5.76, *p* = 0.016). The use of oral hygiene products also differed significantly between the depression group (*n* = 109, 39.1%) and the no-depression group (*n* = 769, 31.1%) (χ^2^ = 7.26, *p* = 0.007). The mean daily tooth brushing frequency was 2.06 ± 1.22 in the entire study population. The percentage of participants who brushed their teeth three or more times a day significantly differed between the depression group (*n* = 109, 39.1%) and the no-depression group (*n* = 831, 33.6%) (χ^2^ = 10.34, *p* = 0.006) (Table 2).

### 3.3. Predictors of Depression in BLSP-Benefit Recipients

Logistic regression was performed to identify the predictors of depression in BLSP-benefit recipients (Table 3). In model 1, age, sex, marital status, education, ADLs, moderate physical activity, economic activity, and self-rated health, which significantly differed in relation to depression in the univariate analyses, were entered as the independent variables and depression diagnosis was used as the dependent variable for a binomial logistic regression to identify the predictors of depression diagnosis in BLSP-benefit recipients. The regression model was statistically significant (χ^2^ = 48.01, *p* < 0.001), and the explanatory power as determined by the Nagelkerke coefficient of determination was 31.7%. Classification accuracy was 89.9%, and a good model fit was established, as the hypothesis that the observed values of the model do not differ from the predicted values was not rejected based on the Hosmer–Lemeshow test (χ^2^ = 10.38, *p* = 0.239).

The predictors of depression among BLSP-benefit recipients were identified as age, sex, education, economic activity, moderate physical activity, and self-rated health.

In model 1, the odds ratio (OR) for the diagnosis of depression was 1.45 in individuals with an older age (95% confidence interval [CI] 1.07–1.97), 1.72 for males as compared to females (95% CI 1.29–2.29), 1.81 for people with elementary school or less education as compared to those with middle school or higher education (95% CI 0.36–0.65). Those who do moderate-intensity physical activity were 53% (OR, 0.47; 95% CI 0.30–0.74), less likely to be diagnosed with depression as compared to those who do not. There was a 64% reduction for those who were economically active as compared to those who were economically inactive (OR, 0.36; 95% CI 0.30–0.72), and 63% reduction for those with good self-rated health as compared to those with poor self-rated health (OR, 0.37; 95% CI 0.22–0.62).

In model 2, oral health practices (oral health checkup in the past year, use of oral hygiene products, tooth brushing frequency) that significantly differed according to depression in the univariate analyses were added to the independent variables used in model 1 (Table 3). In model 2, the predictors of depression were age, sex, education, moderate physical activity, and self-rated health. The OR for the diagnosis of depression was 1.37 for older age (95% CI 1.01–1.87), 1.74 for males compared to females (95% CI 1.29–2.24), 1.66 for those with elementary school or less education as compared to those with middle school or higher education (95% CI 1.21–2.27). Those who do moderate-intensity physical activity were 53% (OR, 0.47; 95% CI 0.30–0.73), less likely to be diagnosed with depression as compared to those who do not. There was a 64% reduction for those with good self-rated health as compared to those with poor self-rated health (OR, 0.37; 95% CI 0.22–0.61).

Oral health behaviors entered in model 2 did not influence the diagnosis of depression (Table 3).

## 4. Discussion

This study aimed to investigate the association between oral health behaviors and depression and the predictors of depression in BLSP-benefit recipients, to provide a basis for developing interventions for depression in BLSP-benefit recipients, who represent a vulnerable social group, by using the sixth and seventh (2013–2018) KNHANES data sets. As predictors of depression among BLSP-benefit recipients, we identified sex, age, education, ADLs, moderate physical activity, economic activity, and self-rated health.

In our study, the risk for the diagnosis of depression was significantly higher among men than women. On the other hand, depressed mood was significantly more prevalent among women in another study on adults using the eighth KNHANES data sets [22], and major depressive disorder was more prevalent among women in a study using the sixth (2014) KNHANES data set [29]. These findings were contradictory to our findings. This was quite different from analyzing depressive mood for 2 consecutive weeks [22] as well as from analyzing the predictors of depression disorder based on a PHQ-9 score ≥ 10 [29]. According to the 2015 Health and Chronic Disease statistics [21], the prevalence of depressive mood among men and women rose from 6.6% and 13.7%, respectively, in 2013, to 13.7% and 16.5%, respectively, in 2015. In particular, the more than two-fold increase in the prevalence of depressive mood among men was notable, a change in depression status by sex and the topic warrants more societal attention and preventive and management measures. Therefore, further large-scale studies are needed to analyze the diagnosis of depression among BLSP-benefit recipients.

In the case of the United States, stressful events such as bereavement, medical illness and substance use disorders, or medication were identified as contributing factors to depression [30]. In Korea, the factors influencing depression were social network satisfaction, self-esteem, perceived health status, socioeconomic status and regular exercise, and cognitive level [31,32] and in the case of Korea, middle-aged adults in their 40s and 50s frequently experience depressive symptoms, based on the 2016 KNHANES data [29]. Therefore, it is necessary to pay attention to depression prevention and treatment intervention strategies for middle-aged and older people.

In terms of education level, people with an elementary school education or less had a significantly higher risk for depression as compared to those with a middle school or higher education, similar to previous findings that the prevalence of depressive symptoms was higher among the less-educated individuals [33]. This discrepancy may be attributable to the fact that our study data were obtained from people medically diagnosed with depression, while the study by Jung, Kim, and Seong [22] and the study by Yang [34] analyzed the experiences of depressive symptoms for 2 consecutive weeks in the past year. The PHQ-9 is a self-reporting instrument; thus, individuals’ responses may be influenced by their demographic characteristics and personalities, which reduces the objectivity of the responses. Hence, continued research on depression in vulnerable social groups is required.

Those who did not engage in moderate-intensity physical activity were at significantly higher risk of a diagnosis of depression. While a direct comparison is difficult, the experience of depressive mood is significantly higher among people who do not regularly walk in one community [34]. In addition, previous findings that walking on 1–2 days a week helps prevent depression in older adults [35] are in line with our findings.

In addition, the risk for diagnosis of depression was significantly higher among economically inactive individuals. Previous findings that being economically inactive is a predictor of depressive mood [34], that the prevalence of depressive mood was higher among BLSP-benefit recipients [34], and that mental health (e.g., depression) was worse with decreasing SES [19] supported our results. Moreover, the prevalence of depressive mood was 6.3% among National Health Insurance subscribers and 13.1% among Medical Aid recipients in a previous study [33], showing a relatively higher prevalence in the economically vulnerable group. Thus, lowering the risk for depression through mental health management among BLSP-benefit recipients appears to be important. The risk for a diagnosis of depression was significantly higher in those with a poorer health self-rating, consistent with previous findings that moderate or very poor self-rated health predicted depression, in a study that used KNHANES data [29]. In another study, by Lee et al., more than half of the adult depression (≥19 years) group had poor self-rated health and self-rated health was a significant risk factor for depression [36]. Moreover, self-rated health was found to influence depression in older adults who receive BLSP benefits [37]. As shown here, self-rated health is an important risk factor for depression.

In the present study, oral health practices, namely oral health checkups in the past year, use of oral hygiene products, and tooth brushing frequency were not significant predictors of the diagnosis of depression. A previous study that used the fourth and fifth KNHANES data also found that tooth brushing and oral examination did not influence depressive symptoms in middle-aged adults [38]. On the other hand, a study using the seventh KNHANES data reported that the risk for depression increased with decreasing tooth brushing frequency, not using floss, and not undergoing oral checkups in adults aged 19 years and over [39]. Although preventive dental treatment was not a significant predictor of depression in our study, compliance with preventive dental treatments was 19.4% in the depression group and 84.5% in the no-depression group, showing that the majority of patients with depressive symptoms do not seek preventive dental treatments. Thus, it is imperative that the government should establish policies to promotes preventive oral care and implements relevant oral health intervention measures for those who receive BLSP benefits.

This study has some limitations. First, this study was a cross-sectional survey using the KNHANES data. Thus, the results do not provide causal implications. Second, self-rated health, perceived oral health status, and moderate physical activity were collected through a self-rated questionnaire. Hence, a possible response bias must be taken into consideration when interpreting the results.

Despite these limitations, however, this study was significant in that it attempted to examine the factors that influence the diagnosis of depression in relation to oral health practices in recipients of BLSP benefits, which is a vulnerable social group, and to examine their problems in more depth using nationally, for many years Korea representative data.

## 5. Conclusions

This study analyzed the data of 2749 BLSP-benefit recipients aged 19 years and over from the sixth and seventh KNHANES data (2013–2018) in order to identify the relationship between oral health practices and depression, as well as predictors of depression in this population with a view to providing a basis for improving both their oral health and mental health. Our study results showed that male sex, old age, low education level, no moderate physical activity, no economic activity, and poor self-rated health predict a diagnosis of depression. Although oral health checkups in the past year, use of oral hygiene products, and toothbrushing frequency were not significant predictors, they did differ between the groups with and without depression. Furthermore, BLSP-benefit recipients with a diagnosis of depression were found not to seek preventive oral treatments. Thus, we propose that the government should continue to promote and educate people on the importance of oral health and should implement strategic interventions to reduce depression in vulnerable social groups. Oral health is closely linked to the living environment, dietary patterns, oral hygiene habits, and knowledge and attitude toward oral hygiene. Since most oral problems can be managed by prevention, programs that promote oral health practices are needed.

Since there are various characteristic symptoms of depression according to age, it is important to properly evaluate them. In the case of depression in old age, it is easy to overlook the symptoms of depression, especially in many cases, which are considered to be a process of physical illness and normal aging. Therefore, it is necessary for medical staff to provide assessment through appropriate screening tools for each age group and education and intervention to prevent depression by age group, for national policymakers to come up with strategies for providing jobs that can enhance self-esteem through economic activities for BLSP-benefit recipients with depression. Furthermore, continued, multilateral research is needed to analyze the factors influencing depression in BLSP-benefit recipients using large sample data.

## Figures and Tables

**Table 1:** General characteristics of subjects with and without depression (*N* = 2749).

Variable	All (*n* = 2749)	With Depression (*n* = 279)	Without Depression (*n* = 2470)	χ^2^ or *t*	*p*
	*n* (%) or Mean ± SD				
Age (years)	56.26 ± 18.02	50.73 ± 22.10	50.33 ± 17.51	20.04	<0.001
20–39	508	32 (11.5)	476 (19.3)		
40–64	1064	142 (50.9)	922 (37.3)		
65–74	584	62 (22.2)	522 (21.1)		
≥75	593	43 (15.4)	550 (22.3)		
Sex				13.39	<0.001
Male	1047	75 (26.9)	972 (39.4)		
Female	1702	204 (73.1)	1498 (60.6)		
Marital status				5.21	0.022
Married	2203	238 (85.3)	1965 (79.6)		
Unmarried	546	41 (14.7)	505 (20.4)		
Education level				23.75	<0.001
Elementary school below	1475	128 (45.9)	1347 (54.5)		
Middle school	318	55 (19.7)	263 (10.6)		
High school	660	73 (26.2)	587 (23.8)		
≥College	296	23 (8.2)	273 (11.1)		
Economic activity				15.99	<0.001
Yes	968	68 (24.4)	900 (36.4)		
No	1781	211 (75.6)	1570 (63.6)		
Daily function				59.30	<0.001
Good	1972	161 (57.7)	1811 (73.3)		
Not good	718	97 (34.8)	621 (25.1)		
Poor	59	21 (7.5)	38 (1.5)		
Moderate-intensity physical activity				8.14	0.004
Yes	169	28 (10.0)	141 (5.7)		
No	2580	251 (90.0)	2329 (94.3)		
Smoking				4.16	0.125
Current Smoker	857	74 (26.5)	783 (31.7)		
Ex-smoker	478	46 (16.5)	432 (17.5)		
Non-smoker	1414	159 (57.0)	1255 (50.8)		
Alcohol drinking				0.70	0.403
Yes	2276	226 (81.0)	2050 (83.0)		
No	473	53 (19.0)	420 (17.0)		
Perceived health status				45.35	<0.001
Good	424	17 (6.1)	407 (16.5)		
Moderate	1074	85 (30.5)	989 (40.4)		
Poor	1251	177 (63.4)	1074 (43.5)		
Perceived oral health status				1.40	0.498
Good	134	10 (3.6)	124 (5.0)		
Moderate	428	41 (14.7)	387 (15.7)		
Not good	2187	228 (81.7)	1959 (79.3)		
PHQ-9 Score	1.17 ± 0.44	1.48 ± 0.69	1.14 ± 0.39	−0.89	<0.001
Minimal, 0–4	2356	178 (63.8)	2178 (88.2)		
Mild to moderate, 5–14	319	69 (24.7)	250 (10.1)		
Severe, ≥15	74	32 (11.5)	42 (1.7)		

PHQ-9, Patient Health Questionnaire-9.

**Table 2:** Differences in oral health behavior according to the diagnosis of depression (*N* = 2749).

Variable	All (*n* = 2749)	With Depression (*n* = 279)	Without Depression (*n* = 2470)	χ^2^	*p*
	*n* (%), M ± SD	*n* (%)	*n* (%)		
Dental visit experience in a year				5.76	0.016
Yes	532 (19.4)	69 (24.7)	463 (18.7)		
No	2217 (80.6)	210 (75.3)	2007 (81.3)		
Using oral hygiene supplies				7.26	0.007
Yes	878 (31.9)	109 (39.1)	769 (31.1)		
No	1871 (68.1)	170 (60.9)	1701 (68.9)		
Preventive dental treatment (per year)				2.71	0.099
Yes	438 (15.9)	54 (19.4)	2086 (84.5)		
No	2311 (84.1)	225 (80.6)	384 (15.5)		
Frequency of tooth brushing (time/day)	2.06 ± 1.22			10.34	0.006
0	370 (13.5)	21 (7.5)	349 (14.1)		
1–2	1439 (52.3)	149 (53.4)	1290 (52.2)		
≥3	940 (34.2)	109 (39.1)	831 (33.6)		

**Table 3:** Predictors of basic livelihood recipient with depression (*N* = 2749).

	Model 1			Model 2		
	Exp(B)	95% CI		Exp(B)	95% CI	
		Lower	Upper		Lower	Upper
Age	1.45	1.07	1.97	1.37	1.01	1.87
Sex ^†^	1.72	1.29	2.29	1.74	1.29	2.24
Marital status^†^	1.05	0.68	1.63	1.04	0.67	1.61
Education ^†^	1.81	0.36	0.65	1.66	1.21	2.27
Daily activities ^†^	0.75	0.55	1.01	0.89	0.63	1.32
Moderate-intensity physical activity ^†^	0.47	0.30	0.74	0.47	0.30	0.73
Economic activity ^†^	0.36	0.27	0.49	0.73	0.54	1.00
Perceived health status ^†^	0.37	0.22	0.62	0.36	0.22	0.61
Dental visit experience in past year ^†^				1.26	0.92	1.73
Using oral hygiene supplies ^†^				1.20	0.90	1.58
Frequency of tooth brushing ^†^				1.10	0.88	1.37

^†^ Dummy variable: sex (1 = Male), Marital status (1 = Unmarried), education (1 = elementary school below), daily activities (1 = less than poor), Moderate-intensity physical activity (1 = yes), Economic activity (1 = yes), perceived health status (1 = good), Dental visit experience in past year (1 =yes), Using oral hygiene supplies (1 =yes), Frequency of tooth brushing (1 = less than 2 times).

## Data Availability

Not applicable.
